# Progress in State Medicine

**Published:** 1903-08-29

**Authors:** 


### Progress in State Medicine.

(*Concluded from page* 364.)

### MILK.

Until [6] proper management of dairy farms and a high standard of cleanliness are insisted upon the practical value of precautions taken in large cities will be small. Clean stables, clean healthy animals, clean milkers and dairy utensils should be insisted upon; the water-supply and the drainage should be certified by the medical officer of health, as also the health of all those employed on the farm. If it were realised that from a single cow's hair hundreds of bacteria can be imparted to the milk, and that a gram of fresh cow manure contains as many as 375,000,000 bacteria, the authorities would insist upon the cow's hind-quarters being clipped, and their udders washed with clean water and dried with a clean towel before the operation of milking commenced. In order to bring pressure upon the farmers to be clean, it would be essential to have a bacterial standard of purity for the milk (to vary, perhaps, according to the season of the year), but which should be insisted upon by the dealers to whom the milk is supplied. With respect [7] to the infantile mortality in relation to milk supply, the most unfavourable feature in the sanitary history of the last 40 years is the fact that it has not been reduced, but has in fact become on the average higher than it was. Comparing 1851–60 with 1896–99 the infantile mortality has increased about 2 per cent., while the death-rate from all causes together has declined about 21 per cent. The distribution of infantile mortality shows that it is much greater in counties in which urban populations predominate, being especially great in the counties in which mining and manufacturing industries are chiefly found. Under the head of diarrhœa and enteritis the mortality is more than seven times as great in the town as in the country. The importance of artificial feeding in increasing infantile mortality cannot easily be exaggerated. Rotch [8] explains that the purpose of the milk laboratory established in Boston (U.S.A.) in 1891 was to insure a clean, constant, and reliable milk supply, and to provide a place where different combinations of milk may be put up, according to the prescriptions of physicians, with accuracy and under such conditions of cleanliness and asepsis as to insure the best possible food for infant feeding. English cows not being housed so much during the year, and therefore feeding more commonly in the pastures, have less regularity in the quality of their food than occurs with American cows. This, though in a general way desirable, is not conducive to the best milk for infant feeding, as the milk is not so stable as when the food is unvarying. Cows should be groomed in clean sheds away from the stable; dry cleaning being preferable to wet. As nearly an ideal cow-house as possible, should be provided. A barn is needed for cows which are to be used for infant feeding, which will allow each cow at least 1,200 cubic feet of fresh air. A microscopical examination is made of the milk for detecting the reappearance of colostrum corpuscles, pernicious micro-organisms, pus corpuscles, more than five of the last to each field given by a $\frac{1}{12}$ oil immersion lens contra-indicating the milk for the time being for infant feeding. The milkers should be frequently examined by a physician to see if they are diseased in any way. Sterilised milk stools of metal should be used, and sterilised receptacles for receiving the milk. It has been found better to milk the cows in their stalls, where they sleep, and where they feel at home, rather than in a special milking-room. Immediately after milking the milk should be brought in closely covered receptacles to a room at least some hundreds of yards away from the barn and milk-house. The milkers, however, should not be allowed to go into the milk-rooms, and a separate set of men should take care of the grooming of the cows. The dairymen should be healthy, intelligent, and clean, and no one else allowed in the milk-room. Richards[9] thinks that the purchaser has the right to expect that the purchased milk is of average nutritive value, containing all its fat and without water; that it has been obtained from healthy cows, and is clean and uncontaminated. The rights of the purchaser in respect to the nutritive quality of milk are reasonably protected by the Food and Drugs Act, but the requirement that milk should be obtained from healthy cows offers more difficulty. Article 15 of the Dairies, Cowsheds, and Milkshops Order, 1885, provides that milk from diseased cows should not be mixed with other milk, or be used for human food, or if unboiled used for the food of animals. The term "diseased cow" is, by the principal Act and the Amending Order of 1899, limited to cattle suffering from cattle-plague, pleuro-pneumonia, foot and mouth disease, or "such disease of the udder as shall be certified by a veterinary surgeon to be tubercular." He finds fault with this last condition, as apart from the vexed question of the danger of tuberculous milk, there can be no reasonable doubt that it should be unlawful to sell for human food milk containing pus, and possibly teeming with the micro-organisms of suppuration. Furthermore, the Amending Order omits to carry out the most important recommendation of the Royal Commission on Tuberculosis—namely, the compulsory notification of every disease of the udders of cows. With regard to the effect of regulations as to air-space, he is not at all sanguine that they have much effect in improving the health of cattle. Again, mere cubic space without thorough means of ventilation will do little to improve the health of cows. Better results would follow by convincing the farmers that it is to their own interests to keep their cows on the open-air system. It is also suggested that the milk supply of cows, constantly maintained under healthy conditions, is not seriously, if at all, diminished by cold, and that any shortage is compensated by the much greater cleanliness of the milk, the healthiness of the cows, and by the less expense and trouble in looking after them. He thinks it might be practicable to obtain powers whereby it was made penal (1) to expose for sale milk containing more than a certain proportion of dirt; (2) to sell milk that had not been properly cooled immediately after milking; (3) to sell milk that could not be boiled without coagulating within six hours after purchase.

[6] Ibid., April 18, 1903. [7] Ibid., May 30, 1903. [8] Public Health, May, 1903. [9] Ibid.

